# Healthcare professionals’ perceptions of the contributions of rehabilitation coordinators to patients in Swedish psychiatric care: a qualitative descriptive study

**DOI:** 10.1186/s12888-024-05895-w

**Published:** 2024-06-12

**Authors:** Åsa Andersén, Beatrice Carpentsier, Erik Berglund, Maria Carlsson

**Affiliations:** 1 Department of Public Health and Caring Sciences, Uppsala University, Box 564, Uppsala, SE-751 22 UPPSALA Sweden; 2 Division of Insurance Medicine, Department of Clinical Neuroscience, Karolinska Institutet, Stockholm, SE-171 77 Sweden

**Keywords:** Mental disorders, Sick leave, Rehabilitation coordination, Return-to-work, Vocational rehabilitation

## Abstract

**Background:**

Rehabilitation coordinators have gradually been introduced into Swedish psychiatric care to support individuals on sick leave to return-to-work or enter work.

**Aim:**

To explore healthcare professionals’ perspectives on the contributions a rehabilitation coordinator can make to patients in psychiatric care.

**Materials and methods:**

A descriptive qualitative design was used, and data were collected through interviews. Twelve healthcare professionals in psychiatric care participated in individual semi-structured interviews. Data were analysed using thematic analysis.

**Results:**

An overarching theme evolved: “The rehabilitation coordinator promotes security and reduces stress in the vocational rehabilitation process”, based on two themes: (1) “Adaptations and support based on the patient’s needs” and (2) “Rehabilitation coordinator efforts as relevant for care”. The themes, in turn, consist of six subthemes.

**Conclusions:**

This study showed that healthcare professionals perceived employment as important for patients’ health and well-being. Therefore, the rehabilitation coordination efforts were not only seen as beneficial for addressing patients’ challenges and needs in managing the vocational rehabilitation process but also as an integral part of the patient’s care.

## Background

Mental disorders are an extensive problem and a public health challenge in Europe [1]. These conditions entail disturbances in thinking, emotional regulation or behaviour, and can cause significant limitations in various aspects of life [1]. In Sweden, mental disorders are the main cause of sick leave [2]. Except that sick leave due to mental disorders tends to becoming long-lasting [3, 4], return-to-work (RTW) rates are lower, compared to other diagnosis groups. There is also a higher risk of being on sick leave again [3, 5]. A previous systematic review revealed that individuals with mental disorders and chronic diseases have an increased risk of transitioning from paid work to sickness benefits (former disability pension) and unemployment [6]. Of those individuals who become unemployed, it can be difficult to reintegrate back into the workforce, and also to receive the support they need to make this possible [7]. In addition, individuals with mental disorders also have a lower rate of employment in the labour market in general [7], resulting in the risk of being excluded from working life.

A challenge in the field of rehabilitation is to maintain a person’s work ability (if there is any) at a level that enables the individual to work to some degree despite illness or impairment [8]. This has many benefits for individuals and society, as work, in addition to the financial resources it provides, contributes to one’s identity, social status, and participation in society [9]. Furthermore, work can bring a sense of meaning and satisfaction in life [10]. Although work is generally important for health and well-being [9], sick leave of a limited duration can be necessary for healing and recovery. However, research shows that long-term sick leave can have negative effects on individuals, reported as negative consequences on their economic situation, ability to perform leisure activities [11], further deterioration of health [12–14], passivity and lack of motivation [12], thus resulting in extended sick leave [15]. Other negative consequences of sick leave include a sense of loss of independence, being stigmatised, and a change in self-perception [14]. It is, therefore, important to find ways to avoid the risk of individuals ending up in this negative development.

In addition to the fact that it can be difficult for those with mental disorders to obtain work [7], RTW after sick leave can be a complex process, involving factors other than the patients’ medical condition [16]. Several actors may be involved in the patients’ vocational rehabilitation (VR) [17, 18], including healthcare providers, employers, the Public Employment Service (PES), the Social Insurance Agency (SIA), and the municipality, each with different responsibilities [19]. Inadequate collaboration among these actors can constitute obstacles to a patient’s ability to RTW/entry into work [20]. VR can briefly be described as multidisciplinary efforts (medical or nonmedical), intended to enable individuals with illness or injury to RTW or prevent loss of work. These efforts may include assessments (e.g. of function and work capacity), coordination, health promotion to support RTW, and planning, among others [21]. The aim of VR is to optimise work participation via engagement (entry into) or re-engagement in work [22].

In Sweden, rehabilitation coordinators (RCs) have been introduced into the healthcare system to streamline patients’ sick leave process [23]. Since 2020, the Swedish healthcare services have been obligated to offer patients on sick leave rehabilitation coordination, if needed, to support their RTW or entry into working life [24]. The purpose of RCs is to work strategically and intensively to help patients in achieving their work-related goals [24]. RCs’ functions have developed over time and include three main tasks: provide support to patients in their VR process, manage coordination internally (within the healthcare system) and externally (with i.e. the SIA, PES and the patient’s employer). Coordination is not directly seen as care or treatment; instead, it involves investigating, connecting, and facilitating cooperation. RCs can also map the patient’s rehabilitation needs and create a plan for the VR process [25]. The main groups receiving support from RCs are those with mental disorders and musculoskeletal disorders.

Initially, RCs were introduced in primary care, where their function has been developed and evaluated, showing positive effects on patients with mental- or musculoskeletal disorders [25]. Patients who received support from RCs had a lower rate of sick leave, ended their sick leave more quickly, and had fewer instances of repeated sick leaves compared to a control group [26]. However, the same effect could not be seen in patients with both musculoskeletal- and mental disorders. Regarding the effect of RC efforts, previous international studies have shown mixed results, with some studies showing a positive effect on RTW [27], while other studies indicated a delay in RTW [28, 29]. In another study based on longitudinal data, employees with reduced work ability who received support from RCs (employed by the employer) were found to have a higher risk of sickness absence but a lower risk of later disability retirement compared to employees who did not receive such support [30]. Although the overall role of RCs is to facilitate individuals’ RTW and support communication and cooperation among different actors involved in the rehabilitation process [29, 31], the working methods and efforts used may vary [32]. It is, therefore, unclear whether the coordinators involved in the RTW process, as described in the international research (often called RTW coordinators), usually placed in companies or insurance agencies, correspond to the RC function that exists in Swedish healthcare. In addition, study designs, length of interventions, and diagnoses can differ, as can the length of sick leave and current employment status of study participants.

Qualitative studies conducted in primary care show that access to a RC can mean many benefits for the patient; for example, a sense of security and stability throughout the rehabilitation process [33]. RCs are also described as having a positive impact on physicians’ and other healthcare professionals’ (HCPs) work in sick leave and rehabilitation, as they can facilitate cooperation with external actors [34].

In recent years, RCs have gradually been introduced into Swedish specialist care, e.g. psychiatric care. However, research on RCs in psychiatric care is scarce, as well as the overall research regarding the RCs’ function. Previous studies have mainly focused on patients’ perspectives regarding outcomes and experiences in relation to RCs [33, 35] and explored the RCs’ perspective [32, 33, 36]. The aim of this study was to explore HCPs’ perspectives on the contributions RCs can make to patients in the psychiatric setting. This perspective was considered important because it focuses on how the RC function can complement other care related efforts for patients provided by HCPs. This approach also makes it possible to identify advantages and disadvantages of RC interventions that patients may not notice themselves within the psychiatric care process, but which may be recognised by HPCs in their clinical practice.

## Methods

### Study design and setting

The study has a descriptive qualitative research design with data collected through semi-structured interviews with HCPs. This method was chosen because of its suitability in the study of individuals’ thoughts and experiences [37]. The present study was conducted alongside a randomised controlled trial (RCT), with an intervention group receiving support from a RC compared to a control group receiving treatment as usual (TAU). It was carried out in psychiatric care in central Sweden. The RCT was conducted at two care units for patients with moderate-to-severe affective and/or moderate-to-severe anxiety disorders, aged 25–64 years. The participants included in the RCT were on sick leave (could be employed or unemployed), or receiving disability benefits (i.e. temporary compensation for reduced work capability due to illness, for individuals aged 19–29 years). The RCs worked in accordance with the mission outlined for RCs in the Method book for coordination of sick leave and rehabilitation in healthcare [25]. The method is organised across different levels; however, not all levels were included in the intervention. The RCs had education in social sciences and mapped the patients’ rehabilitation needs, focusing on their ability to RTW/entry into work or studies, through interviews. By mapping out the patient’s rehabilitation needs, a rehabilitation plan was established, focusing on RTW/entry into work or studies. Throughout the rehabilitation process, the RC served as a contact person for the patient, offering individually tailored support and initiating and coordinating contacts within and outside of healthcare services, as needed for the patient’s rehabilitation process. The intervention study has been described in an earlier published study protocol [38].

### Participants and recruitment

Participants were recruited from two psychiatric care units where the intervention study was carried out. The RC intervention was first introduced at one unit at the end of 2018, and at the other unit in spring 2021, and then proceeded in parallel. All HCPs working at these units were invited to take part in the study. They received a letter and/or an e-mail with information about the interview study and were asked about interest in participating in an interview. The department heads at the respective units delivered the information. To obtain a variation in the sample, including a representation of different HCPs, and both men and women, the department heads sent reminders via email.

Subsequently, HCPs had to contact the researchers themselves to register their interest in participating in an interview. They received information that participation was voluntary, that they could withdraw from the study at any time, and that all data would be treated confidentially. Participants who gave their written consent to participate in the interview and to audio recording were included in the study. Recruitment of participants ended when the authors determined that no new themes were emerging during the interviews.

### Data collection

A total of 15 interviews were conducted with physicians, a psychiatric aide, nurses, psychologists, and social workers. Two interviews were excluded from the analysis, as the participants lacked knowledge about and experience of working with RCs. One interview was excluded due to poor sound quality, with no ability to hear what was being said on the recording. Thus, twelve interviews with HCPs were included, with two men and ten women (see Table 1). The interviews were carried out between June 2019 to October 2021, with one conducted by the first author (Å.A.) and the rest by the last author (M.C.), who has training and experience in qualitative interviewing. The interviews were performed individually, either face-to-face at the clinics or by phone. They ranged from 23 to 47 min in duration. All interviews were audio-recorded and transcribed verbatim. The researcher used a semi-structured interview guide during the interview, based on HCPs’ perceptions about their own role when working with patients on sick leave, their role in supporting RTW/entry into work or studies, what it means to have a RC at the clinic, and the contributions RCs can make to patients. To obtain a complete and detailed picture of the different activities described in the Method book, the interviewer prompted participants with probes if those activities were not mentioned by the interviewee. The present study only focuses on HCPs’ perspectives on the contributions that RCs can make to patients at the clinic.

**Table 1:** Overview of included informants’ healthcare profession and gender

Profession	Gender
Nurse	Female
Nurse	Female
Psychiatric Aide	Female
Physician	Male
Physician	Female
Physician	Male
Psychologist	Female
Psychologist	Female
Social worker	Female
Social worker	Female
Social worker	Female
Social worker	Female

### Data analysis

Data analysis was performed using inductive thematic analysis with a semantic approach, according to the six-step process outlined by Braun and Clarke [39], without following any theoretical framework. Thematic analysis is a flexible and theoretically independent method widely used for analysing qualitative data [39]. Themes and subthemes were revised several times and checked against each other and the original dataset, data extracts, and codes. The process was interactive, going back and forth with several revisions; some adjustments were even made during the final steps; see Table 2 for an example of the data analysis process.

The analyses were mainly performed by the first (Å.A.) and second (B.C.) authors. The first step was carried out independently, while the remaining steps were carried out together. Discussions concerning the coding and themes were held regularly throughout the analysis process. The last author (M.C.) peer-reviewed the analysis several times during the analysis process. Joint discussions were held to reach a consensus on the themes and subthemes.

The COREQ checklist was used to ensure quality by reporting important aspects of the research process [40].

**Table 2:** Examples of data analysis from data extract to themes

Data extract	Codes	Subthemes	Theme
But somehow, when they (the patients) falls, the RC* is somehow still standing there offering more alternatives. Let’s try again now… (Social worker)	The RC is still there and doesn’t give up	Individual guidance and practical help	The RC promotes security and reduces stress in the vocational rehabilitation process
They (the RC and the patients) can meet more often, they can have phone contact since the RC can have a different focus. (Physician)	The RC is available to the patients	Ongoing contact with a clear focus	

* rehabilitation coordinator (RC)

## Results

**Fig. 1:**
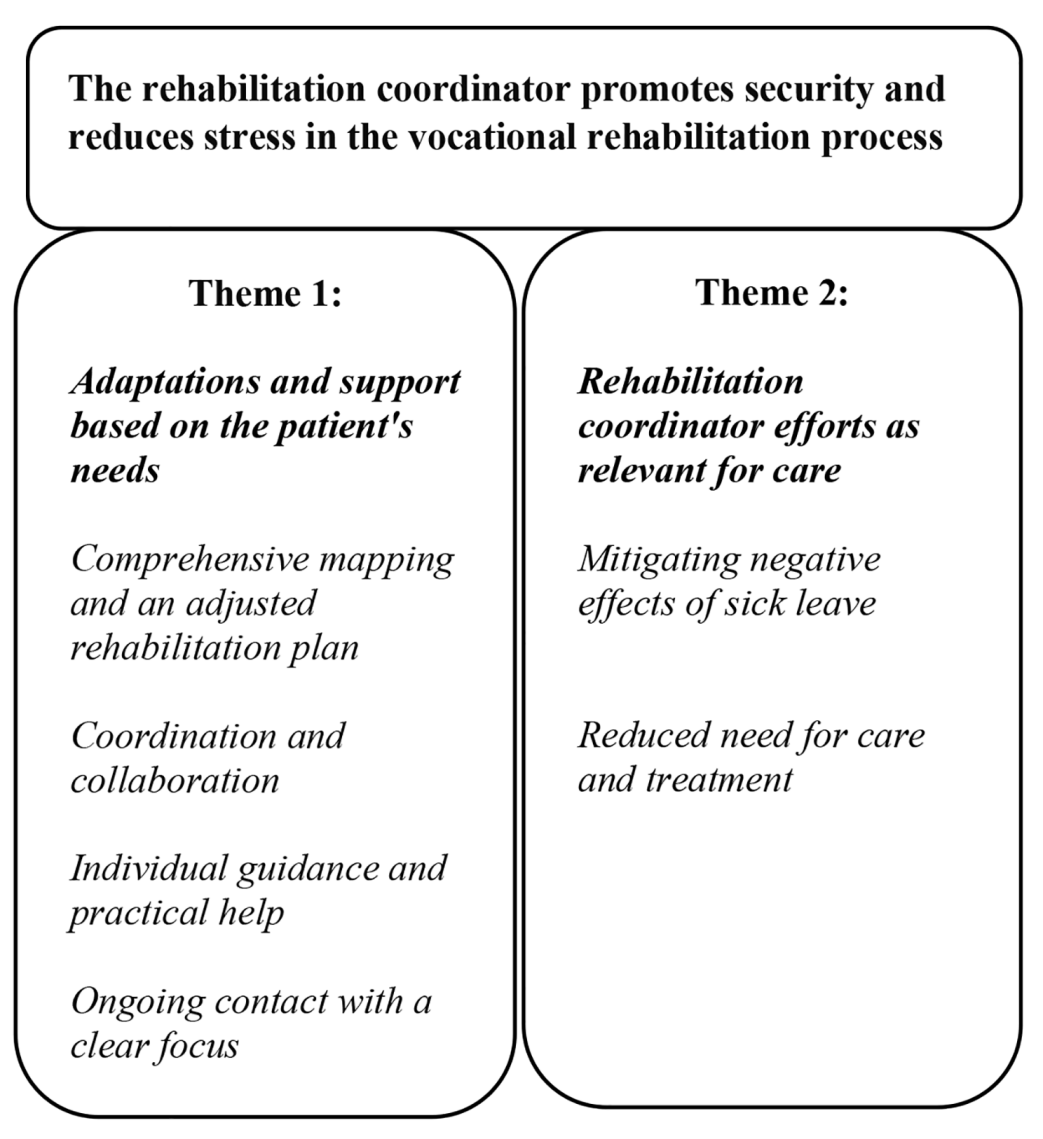
Overaching theme, themes and subthemes

An overarching theme evolved: *“The rehabilitation coordinator promotes security and reduces stress in the vocational rehabilitation process”*, based on two themes: (1) *“Adaptations and support based on the patient’s needs”* and (2) *“Rehabilitation coordinator efforts as relevant for care”.* The themes, in turn, consist of six subthemes. The relationship between the overarching theme, themes, and subthemes is illustrated in Fig. 1.

The HCPs were positive about the introduction of the RC function at the care units and described that there is a need for rehabilitation efforts, with a focus on work/employment, within the patient group. Previously, the ability to offer such efforts had been limited. Given that some patients are on sick leave for longer periods, previous efforts related to employment were considered substandard. Many patients were perceived to require help with RTW/entry into work or studies, as they struggled to manage this process on their own, and their mental health often led to passivity. The RC was, therefore, seen as capable of providing more comprehensive support to patients during the rehabilitation process and motivating the patient to engage with it.

### Theme 1: Adaptations and support based on the patient’s needs

This theme describes HCPs’ perception about the importance of different RC efforts, with regard to patients’ difficulties and needs in promoting the VR process and patients’ ability to RTW/entry into work or studies. The RC was described as ‘holding together’ the VR process and ensuring that it moves forward.

#### Comprehensive mapping and an adjusted rehabilitation plan

The RC’s mapping of the patient’s rehabilitation needs, with a focus on rehabilitation as needed for work, was seen as positive. Patients were often considered to have specific needs which had to be taken into account when planning for RTW/entry into work or studies; for example, patient-specific needs related to recovery. This was viewed as contributing to the patient’s personal commitment to subsequent rehabilitation efforts.

*“I think that it is very important and extremely positive, since it often happens that people otherwise get sorted into some kind of category due to their diagnosis or length of sick leave and… this intervention is usually suitable for this type of patient.”* (Nurse).

The mapping was considered to be capable of increasing the possibility of finding an occupation that met the patient’s needs, which was seen as a prerequisite for the patient’s progression in the VR process. The RCs work was described as being based on a holistic view, where various factors are taken into account, which may be of importance to the patients’ ability to either RTW or begin working. This could, for example, include initiating contact with other HCPs or actors as a first step in the patient’s VR process.

The RC’s methods of working were perceived as flexible and able to make adjustments according to the patient’s wishes and needs. HCPs also believed it was important that patients felt involved in the planning process and that the RC listened to them. This allowed the patients to have trust in the RC and in the efforts being proposed. Furthermore, both the mapping process and the support provided by the RC were seen as contributing to a sense of security for patients throughout the VR process.

*“So I think that the mapping is at least as relevant as this general support function in order to make the patient feel more confidence in the rehabilitation process, because it might help us to actually find a place of employment where they would do well”. (Physician).*

Having a particular rehabilitation plan for RTW/entry into work, hereinafter referred to as a VC plan—which the RC and the patient draw up together, after the mapping had been carried out—was believed to increase the focus on the patient’s needs when it came to rehabilitation efforts. Some HCPs believed that involving an occupational therapist was crucial for accurately assessing the patient’s functional ability and developing a perfectly good rehabilitation plan. RC support was viewed as contributing to a structured and well considered planning of the VR process, with a focus on establishing long-term and realistic goals for the patient, based on their individual conditions.

*“What will work for this person, because it is quite easy to implement something that, yes…on paper increases the capacity for a short time but does not work in the long-term or does not lead anywhere”. (Nurse).*

The VR plan was found to make it easier for the patients to follow the VR process step by step and thereby experience the process as more manageable, with the opportunity to prepare for each new step in the process. Having a VR plan included in the sickness certificate was also perceived to facilitate contact with SIA for some patients.

*“Or it is positive for the patient partly because there is a plan included on the certificate of illness, which is usually helpful in contacts with the Social Insurance Agency”. (Nurse).*

#### Coordination and collaboration

Patients were often described as having difficulties with coordination, planning, and establishing and maintaining contacts with several actors at the same time, and struggling to cope with this on their own. Interacting with different actors could also induce stress among patients. The possibility of support and help from the RC with coordination and collaboration was, therefore, described as significant for patients and as something that patients appreciated. The RC could instruct and prepare patients before they contacted, for example, the administrator at SIA, and if necessary, the RC could also initiate this contact for them.

*“And I think that many get stuck in a state of stress as soon as they think that now I need to contact my case officer, that they need someone to help structure it all”. (Psychologist).*

The RC had the opportunity to participate in various collaborations. HCPs described that meetings with, for example, PES or SIA could be experienced by patients as frightening. In addition, the RC was viewed as having the ability to advance patient’s cases in situations when the patients themselves were unable to do so. Thus, the RC was seen as a patient representative in these contexts. Furthermore, the RC could also ensure that the patient’s rights were not violated during the VR process. Altogether, the RC’s support and participation in coordinating and collaborating during the VR process were described as contributing to an increased experience of security among patients.

*“…that it’s so difficult when you are already feeling bad and then you have to sit...so like, I think it’s actually a great relief to have someone come with them who can help them (the patients)”. (Nurse).*

#### Individual guidance and practical help

The RC was described as both being able to guide patients during the VR process, and also collaborating with patients to investigate and identify different ways of moving forward. HCPs also perceived that patients they met felt supported by the RC in finding a way forward that took into account their individual conditions and interests.

*“So it’s probably very good to not have staked out too many steps in advance but instead to perhaps look at, this opportunity and that opportunity…which one shall we do first, okay… And anyway my experience is that RCs keep doors open and don’t restrict themselves to following one single plan. And I think that is very important”. (Nurse).*

Through the RC, patients gained insights into available and adapted interventions, leading them to discover new possibilities. Having a RC who remains with the patient in the event of any setbacks and who does not give up, and suggests other paths that the patient can try to advance in their VR process, was deemed significant. HCPs believed that the RC can help instil hope in patients, help them set goals, and explore options to move forward in the VR process.

*“Because I think there is some significance in instilling hope in the existence of a future. You won’t always just lie here in the dark and you won’t be a disability pensioner at the age of 35”… (Social worker).*

The RC provided information and knowledge to the patients about the VR process, as well as access to various forms of support and current regulations. This was seen to contribute to patients feeling more secure in the process and becoming more equipped to navigate it. In addition, the RC was described as being able to offer practical and administrative help, for example, helping to complete the right forms, and filling in certificates and applications of various kinds.

*“The patient might want to study or something like that, but they feel that they can’t manage it at all because they have issues and then the RC can provide them with information about how to get support. For example, it might be someone with autism who is eligible for special accommodations that are helpful”… (Physician).*

#### Ongoing contact with a clear focus

The RC’s specific work, with its focus on employment/RTW and the opportunity to provide regular support to the patient over time, was described as important. There was a perceived need for a function that could oversee the entire VR process. This was viewed by HCPs as activating empowering patients and as something that could actually move them forward in the process, leading to real change.

*“Yes, I believe that having one rehabilitation-focused contact contributes something more than having it somewhat to the side of other contacts or working on it sporadically, because I believe that it evokes more, like…it activates these people more to have one specific contact for this, where there is not so much else involved, because then you can really make progress”.* (Psychologist).

The RC was described as being available to support patients throughout the VR process, accessible to patients through personal meetings or over the phone. The RC was viewed as a personal contact, which was considered significant, as it made the patients feel secure in their situation. Creating this sense of security and alliance with the patient was perceived to take time, but judged as important to achieve success, and to help the patient cope with this changing process to ultimately achieve better health in the end. The regular contact with the patients was also viewed positively, as it was considered to create prerequisites for follow-up, allowing the RC to quickly identify any emerging patient needs and take action accordingly, thereby avoiding issues from lingering over time.

*“Yes, or that there is also quite regular contact with the patients and that in those circumstances, things may come out…and it becomes a bit clearer that there is an impact from the patient flipping their day/night body clock and needs like, yes…has such difficulty sleeping that something maybe needs to be done about that in order to be able to get going to job training”… (Nurse).*

### *Theme 2: Rehabilitation coordinator efforts as relevant for care*

This theme highlights HCPs’ perception of the risks associated with a passive sick leave process and the importance of employment with regard to the patients’ health and well-being, whereas the efforts of the RC and VR are proposed to be a part of the patients’ care and treatment. The RC’s knowledge and involvement in the VR process was considered to lessen stress and anxiety in patients, thus reducing the need for other HCPs to address these issues.

#### Mitigating negative effects of sick leave

Employment in any form was described as able to contribute to improvement in mental health in the target patient group. For patients, employment could, among other things, provide a sense of purpose and structure in everyday life. This was something the HCPs felt that many of their patients needed. Furthermore, employment was seen as a means of normalisation, partly through self-sufficiency and coherence and because patients’ friends and acquaintances usually include working individuals.

*“They (the patients) need employment for the sake of their health; they need routines. We know that this is something that provides support and security, that you have a place where you can go to meet people so that you avoid the isolation that many patients end up experiencing”. (Physician).*

HCPs believed that the ability to offer VR efforts was a prerequisite for patients to have the opportunity to secure adapted and meaningful employment. It was noted that too little consideration is given to this aspect in the planning of patient care and treatment. Since employment was deemed crucial for improving patients’ mental health, it was considered beneficial to discuss this early in the sick leave process with all patients. Accordingly, HCPs would like to see RCs integrated into patients’ planned care, in parallel with other efforts. Enabling early interventions was deemed particularly important for younger patients to avoid negative effects on health.

*“There is also an aspect that…many also feel worse because they don’t have anything to do all day; they go around in circles at home and get even more depressed”. (Nurse).*

#### Reduced need for care and treatment

More resources and increased support for patients in the VR process were perceived as leading to reduced stress and accordingly, a decreased need for care and treatment. Therefore, RCs were seen as a necessary effort. HCPs also saw advantages in that the RC could assist patients in contacts with different authorities. Often, these interactions involved questions connected to the patients’ compensation, which could cause anxiety and stress, thus having a negative impact on their mental health. This, in turn, required attention from the healthcare team. Consequently, HCPs might spend a lot of time dealing with such issues during patient treatment; for example, when sick certificates were not approved and modifications were required. Access to a RC, with knowledge in the field— including these contacts—was therefore considered helpful in facilitating patients to navigate these situations.

*“…it’s always like supplementary documentation is required for their certificate of illness; there is always something that doesn’t go smoothly and it becomes very stressful and they (the patients) might not receive their money on time. This becomes an enormous source of stress, which often becomes quite a lot to handle, like even as a part of a treatment contact for them, which is understandable. If it is your income that is basically at stake, then it becomes very stressful and I think that many perceive the Social Insurance Agency as very harsh, difficult to cooperate with, and such. I mean, it makes it so much easier if you have someone that you can trust, who knows how everything should be done”. (Psychologist).*

## Discussion

The study explored HCPs’ perspectives regarding the contributions RCs can make to patients in psychiatric care. An overarching theme emerged: *“The rehabilitation coordinator promotes security and reduces stress in the vocational rehabilitation process”*, comprising two main themes: (1) *“Adaptations and support based on the patient’s needs”;* (2) *“Rehabilitation coordinator efforts as relevant for care”.* The themes, in turn, were divided into six subthemes.

The HCPs described how patients could receive support from a RC in various ways, guiding them through the VR process and making them feel safe. Similar results were observed in a study [41] with individuals on sick leave with different types of diagnoses, who participated in a VR intervention. In this intervention, the professionals (from the PES, the SIA or the municipality worked together and had fewer clients compared to ordinary service) involved in the VR process, worked closely with the individuals. These individuals reported that having accessibility and continuous contact with the same professional over time instilled a sense of security. The individuals also highlighted the importance of receiving support in various forms, such as assistance from professionals in filling out forms and managing medical certificates, etc., which provided them with relief [41]. This kind of administrative support provided by the RCs to patients was also described by the HCPs in our study.

HCPs stated that it could be difficult for patients to initiate contact but also to manage several contacts and coordinate these, which could cause them stress. Support from the RC in handling these contacts was therefore seen to be of importance for the patients. The importance of coordination has also been emphasised in another study involving patients with depression/bipolar disorder [42], where it was noted that coordination helped ensure that individual patients did not fall through the cracks. Additionally, contact with different actors was described as stressful and could hinder the development of a trusting relationship. In our study, the opportunity for RCs to participate in meetings, for example, with the SIA, was deemed valuable, as patients might find such meetings daunting and stressful on their own. Ståhl et al. [43] describe the importance of considering contact with the SIA (and also the individual’s employer, if there is any) in such situations, as the conditions for equal dialogue may differ compared to healthcare because the SIA has influence over the patient’s sickness compensation. According to the HCPs, the RC could offer patients advice and prepare them before they had contact with different actors. The significance of follow-up by the RC was mentioned by the HCPs in our study, since this was perceived to be important in ensuring that the VR process proceeded as planned. The regularity of contact with the patients, in combination with the RC’s clearly defined mission, was perceived as important for engaging patients and progressing in the VR process. These aspects are all relevant for keeping the patient motivated and streamlining the sick leave process, which are important goals of the RCs’ overall mission [25].

HCPs perceived that RCs’ mapping of patients’ rehabilitation needs created better conditions for patients to progress in the VR process and RTW/entry into work. This mapping was also considered an important basis for the VR plan. Involvement of a RC in designing a VR plan was shown in a quantitative study [35] to be associated with experiencing more support in the VR process. In our study, the VR plan was considered valuable, as it allowed patients to follow the VR process step-by-step, concentrating on their specific VR needs. The importance of receiving individually adapted VR based on the individual’s needs is also expressed in a previous study [42], where it was deemed critical for successful VR. Individuals on sick leave have also been described as perceiving that the VR plan gave them a better understanding of the VR process, in which they wanted to be actively involved. They expressed a need to understand what was supposed to happen during the VR process, which otherwise created uncertainty and a sense of loss of control [44]. I.

As RC efforts were mainly provided within the primary care before, and the target patient group in the study often had been referred to the psychiatric care, the introduction of RCs enabled patients to receive this kind of support also within psychiatric care. The HCPs in the study highlighted the need for VR efforts focusing on work/employment within the patient group and noted that the ability to offer such efforts had previously been limited. However, employment was perceived to be important for the patients’ mental health, and thus warranted more attention. Therefore, HCPs thought that the RC function would be a positive addition to the patient’s planned care. The necessity of providing efforts to promote RTW/entry into work for patients with affective disorders was also expressed in another study with HCPs (including managers) in psychiatric care. It was also considered to be important to involve someone with knowledge of both the labour market and psychiatry [45]. In our study, the HCPs described how any form of employment could contribute to an improvement in mental health within the patient group. In a quantitative systematic review, the weeks spent in employment by patients with mental disorders (e.g. bipolar disorder, major depression, and schizophrenia) were associated with improvement in terms of quality of life and level of functioning [46]. In another study, conducted by Koletsy et al. [47], individuals with psychotic illnesses, including bipolar disorder, reported that being employed gave them a sense of belonging, purpose in life, more hope for the future, and financial stability [47]. In our study, HCPs described positive benefits of employment, such as providing a sense of context, structure, and normalisation to life, partly through self-sufficiency. It was also noted that the RC could instil hope in patients for a brighter future and a sense that they could achieve success in their VR process.

The HCPs emphasised the benefits of the RC in reducing the need for care at psychiatric clinics by reducing patients’ levels of stress. Otherwise, the HCPs would spend a lot of time addressing stress-related concerns, such as patients’ compensation, during treatment sessions. It has been reported that some RCs advise patients to limit the number of healthcare contacts, which also indicates that RCs may reduce healthcare utilisation [48]. However, previous studies have not found associations between RC interventions and healthcare utilisation [35, 49], warranting further investigation. In the short-term, RCs may increase the use of healthcare by initiating contacts with various HCPs who are needed for a patient’s RTW/entry into work or studies. However, in the long run, there may be a reduction in healthcare utilisation if patients receive adequate rehabilitation and, thus, achieve employment and an improvement in health.

### Methodological considerations

This qualitative study provides knowledge and understanding of the contributions RCs can make to patients in psychiatric care, from the perspectives of HCPs. It provides information about the difficulties and needs of the target patient group and describes how RCs can address these challenges. Some of the findings were also supported by other studies involving individuals with their own experiences of the VR process, although the diagnoses sometimes differed.

Various strategies were used to ensure the trustworthiness of the data [50]. To enable a variation in perceptions and perspectives and a rich description of the research question, healthcare staff with different professional backgrounds and genders were invited to participate in the study. Discussions were held to ensure consistency in the selection of appropriate data extracts, codes, sub-themes, and themes. Examples of the analysis process, from data extraction to theme developments, are illustrated in Table 2. To facilitate an assessment of transferability [51], a detailed description of the context, the HCPs professions and genders, data collection, and analysis processes was provided.

The study has some limitations worth noting. Concerning the data collection, interviews were conducted over a long period of time. This was partly because of interviews being interrupted due to the COVID-19 pandemic, and thereafter being carried out by telephone. However, there were no noticeable changes in the quality of the interviews by phone instead of face-to face, which aligns with a study by Larsson et al. [52]. Another reason for the data collection taking time was the expansion of the intervention to a second care unit (with the same target group of patients) in spring 2021. This could have introduced inconsistencies in the data [51]. Another limitation was that the experience and knowledge of the RCs’ work varied among the HCPs. Consequently, some could provide more detailed descriptions of how the RCs contributed to their work, while others could only speculate on the RCs’ potential contributions. Moreover, two interviews were excluded from the analysis because the interviewed HCPs did not have any knowledge about the RC’s function and how they worked within their care unit. This was surprising, as the intervention had been ongoing for a while. In this case, the RCs’ work was conducted within the framework of an intervention study. However, if this lack of awareness persists when the RC is implemented in routine care, it could be problematic, as this entails a risk that patients will not be offered this type of support. All HCPs at the two care units were invited to participate in the study, although it is unclear how many chose not to participate. We also lack information on whether those who declined participation differ in their perceptions of the potential contributions that RCs can make to patients. A possible explanation for the non-participation could be related to high workload, partly due to the COVID-19 pandemic.

## Conclusions

This qualitative study describes the RC function in Swedish psychiatric healthcare from the perspective and understanding of HCPs in their everyday work with patients. HCPs were positive towards the RC function and described their efforts as beneficial for patients in addressing their challenges and needs. They also perceived that RCs supported patients in managing the VR process, fostering a sense of security and reducing stress. This, in turn, was believed to reduce the need for care from HCPs, who would otherwise have to manage these aspects with the patients. RCs efforts aimed at employment were viewed as likely to increase patients’ opportunities for employment, which was deemed important for the patients’ health and well-being. Although the RCs’ efforts were considered helpful and positive for patients and patient care, there is a need for further examination of patients’ experiences of RCs’ efforts and how they affect patients’ RTW/entry into work or studies.

## Data Availability

The data presented in this study are not available due to ethical restrictions.

## Abbreviations

HCP: Healthcare professional
ICD: The international classification of functioning, disability and health
RC: Rehabilitation coordinator
RTW: Return to work
PES: Public employment service
SIA: Social insurance agency
VC: Vocational rehabilitation
